# Whole brain radiotherapy with adjuvant or concomitant boost in brain metastasis: dosimetric comparison between helical and volumetric IMRT technique

**DOI:** 10.1186/s13014-016-0634-6

**Published:** 2016-04-19

**Authors:** Paolo Borghetti, Sara Pedretti, Luigi Spiazzi, Rossella Avitabile, Mauro Urpis, Federica Foscarini, Giulia Tesini, Francesca Trevisan, Paolo Ghirardelli, Sara Angela Pandini, Luca Triggiani, Stefano Maria Magrini, Michela Buglione

**Affiliations:** Radiation Oncology Department, University and Spedali Civili Brescia, P.le Spedali Civili 1, Brescia, Italy; Medical Physics Department, Spedali Civili Brescia, P.le Spedali Civili 1, Brescia, Italy

**Keywords:** Brain metastases, Dosimetric comparison, Helical IMRT, Stereotactic radiotherapy, VMAT, Homogeneity index, Conformal index, Conformal number

## Abstract

**Background:**

To compare and evaluate the possible advantages related to the use of VMAT and helical IMRT and two different modalities of boost delivering, adjuvant stereotactic boost (SRS) or simultaneous integrated boost (SIB), in the treatment of brain metastasis (BM) in RPA classes I-II patients.

**Methods:**

Ten patients were treated with helical IMRT, 5 of them with SRS after whole brain radiotherapy (WBRT) and 5 with SIB. MRI co-registration with planning CT was mandatory and prescribed doses were 30 Gy in 10 fractions (fr) for WBRT and 15Gy/1fr or 45Gy/10fr in SRS or SIB, respectively. For each patient, 4 “treatment plans” (VMAT SRS and SIB, helical IMRT SRS and SIB) were calculated and accepted if PTV boost was included in 95 % isodose and dose constraints of the main organs at risk were respected without major deviations. Homogeneity Index (HI), Conformal Index (CI) and Conformal Number (CN) were considered to compare the different plans. Moreover, time of treatment delivery was calculated and considered in the analysis.

**Results:**

Volume of brain metastasis ranged between 1.43 and 51.01 cc (mean 12.89 ± 6.37 ml) and 3 patients had double lesions. V95% resulted over 95 % in the average for each kind of technique, but the “target coverage” was inadequate for VMAT planning with two sites. The HI resulted close to the ideal value of zero in all cases; VMAT-SIB, VMAT-SRS, Helical IMRT-SIB and Helical IMRT-SRS showed mean CI of 2.15, 2.10, 2.44 and 1.66, respectively (optimal range: 1.5–2.0). Helical IMRT-SRS was related to the best and reliable finding of CN (0.66). The mean of treatment time was 210 s, 467 s, 440 s, 1598 s, respectively, for VMAT-SIB, VMAT-SRS, Helical IMRT-SIB and Helical IMRT-SRS.

**Conclusions:**

This dosimetric comparison show that helical IMRT obtain better target coverage and respect of CI and CN; VMAT could be acceptable in solitary metastasis. SIB modality can be considered as a good choice for clinical and logistic compliance; literature’s preliminary data are confirming also a radiobiological benefit for SIB. Helical IMRT-SRS seems less effective for the long time of treatment compared to other techniques.

## Background

Brain metastases occur in 30 % of all patients with cancer [1] and the incidence is increasing in relation to the improvement of local control and survival related to new available systemic treatments and to the inability of most drugs to cross the blood brain barrier. Moreover, in Italy, the aging of the population and the better diagnostic accuracy obtained with Magnetic Resonance (MRI) contribute to the increase in frequency of BM diagnosis [2, 3].

The main prognostic factors for patients with BM are the Karnofski Performance Status (KPS) (≥70 or < 70), age (<65 or ≥ 65), controlled primary site and the presence of extra cranial metastasis according to the Recursive Partitioning Analysis (RPA) [4, 5]. Recently, a Graded Prognostic Analysis (GPA) has been introduced in order to consider the histology of the primary tumour to the already known prognostic factors [6–8].

To date, the possible therapeutic approaches to BM are surgery (SUR) [9, 10], whole brain radiotherapy (WBRT) [11] and stereotactic radiosurgery (SRS) alone or in combination [7, 12]. Median overall survival (OS) is 1–2 month with supportive care [13], while after WBRT is 7.1, 4.2, 2.3 respectively for RPA class I, II, III [4]. More aggressive treatments as SUR or SRS alone or combined to WBRT can double OS rates [14] with an acceptable neurotoxicity and quality of life [15–17]. A careful patients selection remains crucial to identify who really benefits of intensified local treatments.

To date, it is widely debated if the linear quadratic (LQ) model is able to predict the biological effects of high fractional doses used for stereotactic treatment in BM. Kirkpatrick suggests that tumour control probability after a single stereotactic fraction is higher than expected according to the quadratic linear model [18]. On the contrary, Brenner argues that the LQ model is theoretically and experimentally validated up to a single fraction dose of 10 Gy [19]. Recently, it has been hypothesized that the introduction in the formalism of a parameter proportional to the cube of the dose (linear quadratic *cubic* - or LQC- model), could result in a better fitting of the resulting dose - local control curve with the clinical data [20]. A revision of 11 studies on SRS in BM has shown a good correlation between the local control rate at 12 months and the biological equivalent dose (α/β 12 Gy), calculated with the LQC model, for fractional doses ranging between 6 and 25 Gy; the analysis concluded suggesting excellent local control rates at 12 months after single-fraction doses higher than 20 Gy as opposed to clearly insufficient results for doses lower than 15 Gy [21].

Some authors suggest that this approach does not allow to exploit all the potential benefits of fractionation in terms of radiobiological redistribution and re-oxygenation. Hall et al. argue that fractionated stereotactic treatments may be more effective than those in single fraction [22]. In addition, tumour cell repopulation and repair of sub-lethal damage may occur when a significant interval between WBRT and SRS is provided [23]. Finally, SRS treatments may require very long treatment sessions, possibly forcing to split them in multiple sessions.

From a dosimetric point of view, the possibility of incorporating the contribution of the boost in larger volumes treated with lower doses is a definite advantage of the simultaneous integrated boost (SIB) compared to SRS [24]. In fact, the stereotactic boost after WBRT adds an unintentional dose to the brain, while SIB technique makes possible to optimize the dose distribution taking into account WBRT and boost simultaneously [25, 26]. Both volumetric modulated arc therapy (VMAT) and helical IMRT are able to deliver high radiation doses to the target with excellent precision, thus preserving organs at risk. Although it is not completely clear how SRS and SIB could be considered biologically equivalent due to the possible limits of applicability of LQ model, a comparative dosimetric and technical analysis of ten cases has been performed, in order to assess benefits or drawbacks of SRS vs SIB and Helical IMRT vs VMAT in terms of cost-effectiveness and appropriateness of different therapeutic techniques.

## Methods

Ten patients with BM in RPA prognostic class I-II were considered for this analysis (in particular 5 with SRS and 5 with SIB). They were selected among the first patients treated with helical IMRT at our Department, in order to identify technical and dosimetric variables relevant in the treatment of BM with stereotactic approach and to compare helical IMRT with VMAT in this clinical setting. All patients had one BM but three of them who had two lesions. Five were treated with SRS after WBRT and the remaining 5 with SIB. SIB was preferred when MRI was available at planning, while SRS boost was postponed after WBRT in order to have MRI to confirm number and dimension of BM. All patients were treated with Helical IMRT. Precautionary dose of 15 Gy as SRS boost and 45 Gy as SIB were prescribed for these cases because they were the first patients treated in this way in our department.

Patients were simulated in supine position using a CT scan (3 mm slice thickness and spacing) over the entire head region; thermoplastic masks were used to immobilize the patients. CT images were co-registered through a rigid protocol with diagnostic MRI (axial T1-weighted gadolinium contrast enhancement sequences).

The whole brain was contoured and considered as WB-CTV; different gross tumor volumes were defined for each metastasis as the contrast enhanced lesion; 5 mm radial and 6 mm cranio-caudal expansion was applied to obtain WB-PTV and 3 mm isotropic expansion to obtain boost-*PTV.*

Organs at risk (OAR) included eyes, lens, optic nerves, chiasm, brainstem and brain parenchyma (excluding BM).

A Helical IMRT dedicated treatment planning system (TPS) and the Pinnacle® V9.2 TPS were respectively used for Helical IMRT and VMAT plans.

VMAT plans were generated for an Elekta Synergy linear accelerator equipped with 1 cm at isocenter MLC. Two or more modulated arcs of 10 MV nominal energy photons beams with maximum dose rate of 600 U.M./min were used (number of arcs used for SRS and SIB VMAT plans are reported in Table 2). Computing grid was of 2 × 2 × 3 mm3. Helical IMRT plans employed the standard nominal energy 6 MV photons beams with either 1.05 cm field width or 2.5 cm field width for SRS boost and SIB respectively. Pitch value was defined 0.1 for SRS and 0.278 for concomitant boost plans. A modulation factor of 2.0 and a computing grid of 1.95 × 1.95 × 3 mm3 (fine calculation) were chosen for optimization process and final dose calculation for both the SRS boost and the SIB. All VMAT plans were coplanar with a single isocenter, even for two lesions ones.

All plans were elaborated by the same planner either for VMAT and Helical IMRT and all of them were re-planned by a second independent expert planner in order to ensure optimization.

For each of the 10 cases, 4 treatment plans were calculated and optimized (one of them was actually delivered).

Plan 1: WBRT (30 Gy/10 fr) followed by SRS boost (15 Gy/1 fr) with Helical IMRT technique

Plan 2: WBRT (30 Gy/10 fr) with SIB up to 45 Gy/10 fr with Helical IMRT technique

Plan 3: WBRT (30 Gy/10 fr) followed by SRS boost (15 Gy/1 fr) with VMAT technique

Plan 4: WBRT (30 Gy/10 fr) with SIB up to 45 Gy/10 fr with VMAT technique.

In the treatment plans with sequential boost, WBRT was provided using two 6MV opposing lateral fields equally balanced. In these cases Helical IMRT and VMAT were used exclusively for the boost. The dosimetric assessment of the organs at risk was performed considering the sum of the doses delivered in the two separate plans (WBRT + SRS boost). Regarding SIB plans, instead, the helical and volumetric techniques were used for the overall plan, to include whole brain and BM. All plans were elaborated in order to obtain the best potentially deliverable plan for an acceptable target coverage, without violations of dose constraints to OAR.

### Statistical analysis

Doses to WB-PTV and boost-PTV were prescribed to 100 % according to the criteria of the International Commission on Radiation Units & Measurements (ICRU). The minimum accepted dose to PTV brain and PTV boost was 95 % of the prescribed dose. None maximum dose limit to PTV boost was established, although it was normally within the 108 % of the prescribed dose.

The doses to the organs at risk were evaluated considering dose-constraints reported in the literature [25–27] (Table 1). Dose constraint to brain without PTV (V12Gy <10 ml) was considered just for the single dose of SRS boost.

**Table 1:** Organ at risk dose constraints used for treatment plans

Organ at risk	Dose constraints	
	SIB	SRS
Brain (without PTV)	Dmean ≤ 32 Gy V35 Gy < 15 % V40 Gy < 5 % V45 Gy < 0 %	V12Gy < 10 cc
Brainstem	Dmax < 35Gy	Excluded
Chiasm	Dmax < 35Gy	Excluded
Optic nerve	Dmax < 35Gy	Excluded
Lens	Dmax < 5 Gy	Dmax < 5 Gy

Doses at 1 ml (D1ml), 2 % (D2), 5 % (D5), 50 % (D50), 95 % (D95), 98 % (D98), 99 % (D99) and the mean dose to the target volume (Davg) and the volumes to 95 % (V95), 99 % (V99) and 108 % (V108) of the prescribed dose were used for the dosimetric evaluation of PTV boost SRS and SIB.

In order to evaluate homogeneity of the dose distribution and the dose conformity, according to the requirements of ICRU 62 [28], three parameters were analysed:

- *Homogeneity Index (HI)* is an objective tool to analyse the uniformity of dose distribution in the target volume. Various formulae have been described in literature for its calculation and the more descriptive formula is HI = (D2-D98)/Dp, where D2 = minimum dose to 2 % of the target volume indicating the “maximum dose”, D98 = minimum dose to the 98 % of the target volume, indicating the “minimum dose” and Dp = prescribed dose. This is the most commonly used formula in the literature. The reason for choosing D98 and D2, to represent the minimum and maximum dose, is that the calculation of true minimum or maximum dose is sensitive to the dose-calculation parameters, such as grid size and grid placement, and the high dose gradient is common in Intensity Modulated Radio-Therapy [29]. HI basically indicates the ratio between the maximum and minimum dose in the target volume and the lower value indicates a more homogenous dose distribution within this volume. Smaller HI values indicate more homogeneous dose distributions and the optimal value is to be close to 0.
- *Conformity Index (CI)*, defined as the ratio between the total volume enclosed in the references isodose (V_RI_) and target volume (TV) (CI = V_RI_/TV) [29, 30]. This index shows the target coverage related to a specific reference isodose (RI) considered as a reasonable limit of acceptability of the treatment plan, in this case the 95 % isodose. If the conformity index is situated between 1 and 2, the treatment is considered to comply with the treatment plan; an index between 2 and 2.5, or 0.9 and 1, is considered to be a minor violation, and when the index value is less than 0.9 or exceeds 2.5, the protocol violation is considered to be major, but may nevertheless be considered to be acceptable.
- *Conformation Number (CN)* is defined as in the next formula: (PTVpi/Vpi) × (PTVpi/PTV), where PTVpi is the target volume which is irradiated with prescription isodose (pi) and Vpi is the total volume of tissue which is irradiated with prescription isodose [29]. The values of this index can range between 0 and 1; it is usually considered optimal if close to 1, but values between 0.5 and 0.7 can also be considered satisfactory.

Finally, time of treatment delivery for each planning was calculated.

The statistical analysis was performed using t-Student test for the comparison of the PTV Boost, HI, CI, CN and time of treatment values. The differences were considered statistically significant if *p* < 0.05.

## Results

Ten patients were considered for the analysis; for a total number of 13 BM, 52 PTV boost were calculated. The site of disease were cortical lobes, cerebellum and deep structures respectively in 10, 1 and 2 BM. Mean WB-PTV was 1631 cc (range 1275–1899 cc) and mean boost-PTV was 14.32 cc (range 1.43–51 cc). More data are shown in Table 2.

**Table 2:** Characteristics of cases

	pt 1	pt 2	pt 3	pt 4	pt 5	pt 6	pt 7	pt 8	pt 9	pt 10
Number of metastases	1	1	1	2	1	1	1	2	1	2
Site	Right frontal lobe	right semioval center	right parietal lobe	Right frontal-parietal lobe and right occipital lobe	Right occipital lobe	right lateral ventricle	Cerebellar	Right occipital lobe and left parietal lobe	Right temporal lobe	Right and left temporal lobe
Whole brain PTV (ml)	1796.12	1899.90	1440.29	1573.23	1581.73	1554.60	1769.70	1840.10	1583.20	1275.46
Diameter max. of metastasis (mm)	20	13	21	20	18	12	30	23	13	33
				35				8		11
Boost PTV (ml)	10.75	7.25	15.23	13.68	11.01	5.77	21.88	12.10	5.28	25.92
				51.01				1.43		4.79
Number of arcs - SIB	2	3	2	2	2	2	2	4	2	6
Number of arcs - SRS	2	2	2	2	2	2	2	4	2	6

Dose constraints to the OAR evaluated (chiasm, brainstem, right and left lens, right and left optic nerve) were all respected without major violations.

In the SRS cases there was no statistical difference between VMAT and Helical IMRT in terms of respect of OAR, considering that they received the dose due to WBRT and they were basically excluded by the dose contribute of stereotactic boost. In the SIB cases, the OAR were generally better respected in terms of Dmax, particularly for chiasm (30.31 Gy vs 32.36 Gy; *p* = 0.002) and brainstem (30.95 Gy vs 33.11 Gy; *p* = 0.013) with Helical IMRT-SIB compared to VMAT-SIB.

The Brain excluded from the PTV volume (Brain-PTV) appear the most critical OAR:

- In the SIB cases there were no major violations in terms of Dmax <32Gy (31.15Gy and 30.96Gy respectively for Helical IMRT and VMAT), V35 < 15Gy (7.65Gy and 4.32Gy respectively), V40 < 5 Gy (3.08Gy and 1.37Gy respectively) and V45 < 0 Gy (0.02Gy and 0.11Gy respectively), without statistically significant differences between the techniques;
- In the SRS cases Brain-PTV exceeded the dose constraints (V12 Gy < 10 ml) in all VMAT-SRS plans (mean 34.10 ml) and in 4 Helical IMRT-SRS plans (mean 9.27 ml), with a statistically significant benefit for Helical IMRT-SRS (*p* = 0.019).

All but six (95 %) of the PTV boosts volumes received at least 95 % of the prescribed dose. The six plans that resulted in inadequate target coverage had been calculated as VMAT-SIB, VMAT-SRS and Helical IMRT-SRS in two cases each respectively. In particular, five of them referred to patients with two BM. Nevertheless, the mean of V_PTVboost_95% was over 95 % of prescribed dose for each kind of calculated plans, without statistically significant differences (Table 3).

**Table 3:** Dosimetric parameters of SRS plans

	Technique	PTV	Brain-PTV	CI	HI	CN	Time (seconds)
Patient		D95% (Gy)/%	V12Gy (ml)				
1	Helical IMRT-SRS	14.5/96.7	9.0	2.22	0.06	0.70	1150
	VMAT-SRS	14.7/98.3	0.00	2.29	0.07	0.50	386
2	Helical IMRT-SRS	13.9/92.7	6.9	1.17	0.11	0.65	1266
	VMAT-SRS	14.8/98.5	36.3	3.07	0.03	0.30	419
3	Helical IMRT-SRS	14.8/98.3	10.7	1.25	0.22	0.80	1188
	VMAT-SRS	14.7/97.7	58.6	3.28	0.03	0.06	375
4	Helical IMRT-SRS A	14.9/99.3	36.2	5.54	0.04	2.53	2712
	Helical IMRT-SRS B	14.6/96.7		1.11	0.06	0.51	
	VMAT-SRS A	15.5/103.3	13.8	1.42	0.12	1.21	543
	VMAT-SRS B	15.5/103.3		1.12	0.13	0.90	
5	Helical IMRT-SRS	14.8/98.7	16.4	1.48	0.04	0.46	1222
	VMAT-SRS	15.0/100	87.8	0.09	0.02	0.51	578
6	Helical IMRT-SRS	14.8/98.9	10.5	1.56	0.04	0.47	1051
	VMAT-SRS	15.0/100	59.6	6.67	0.06	0.36	549
7	Helical IMRT-SRS	14.9/99.0	1.2	1.26	0.03	0.51	1314
	VMAT-SRS	14.7/97.7	26.0	1.35	0.03	0.42	425
8	Helical IMRT-SRS A	13.7/91.0	0.50	1.00	0.13	0.27	2112
	Helical IMRT-SRS B	14.7/98.1		1.54	0.03	0.42	
	VMAT-SRS A	14.4/96.0	16.5	1.26	0.07	0.41	571
	VMAT-SRS B	14.5/96.7		1.70	0.04	0.44	
9	Helical IMRT -SRS	14.9/99.5	0.54	1.57	0.01	0.37	1182
	VMAT-SRS	14.8/98.5	25.1	3.00	0.01	0.33	403
10	Helical IMRT-SRS A	14.5/96.3	0.87	1.20	0.08	0.48	2784
	Helical IMRT-SRS B	14.3/95.1		0.69	0.09	0.38	
	VMAT-SRS A	14.0/93.3	17.4	1.10	0.13	0.34	427
	VMAT-SRS B	14.0/93.3		0.90	0.12	0.41	
	Helical IMRT-SRS (mean)	14.55	9.27	1.66	0.07	0.66	1598.10
	VMAT-SRS (mean)	14.73	34.10	2.10	0.07	0.49	467.60
	p (T student)	ns	0.019	ns	ns	ns	0.000

Legend: Patients number 4, 8 and 10 had two brain metastases, volume and the dosimetric parameters of each lesion (A and B) are reported in the table

The analysis of dosimetric parameters for SRS plans showed that HI was 0.07 for both the techniques (very close to the optimal), CI resulted acceptable in VMAT plans (2.1) and optimal in Helical IMRT ones (1.66) without significant differences. CN appeared better for Helical IMRT than for VMAT (0.66 vs 0.49 – *p* = 0,067) (Table 3).

Regarding SIB plans, HI was 0.03 and 0.08 for Helical IMRT and VMAT respectively (*p* < 0.001), no relevant differences emerged for CI and CN, that resulted within an acceptable range (Table 4).

**Table 4:** Dosimetric parameters of SIB plans

	Technique	PTV	Brain-PTV				CI	HI	CN	Time (seconds)
Patient		D95% (Gy)/%	Dmean (Gy)	V35 (%)	V40 (%)	V45 (%)				
1	Helical IMRT-SIB	44.4/98.6	30.6	2.4	0.85	0.00	1.61	0.02	0.31	980
	VMAT-SIB	44.3/98.4	31.5	3.0	1.03	0.04	1.83	0.07	0.67	277
2	Helical IMRT-SIB	44.8/99.6	31.5	12.6	5.9	0.13	4.79	0.02	0.49	247
	VMAT-SIB	45.1/100.3	31.3	3.6	1.14	0.10	2.12	0.09	0.74	193
3	Helical IMRT-SIB	43.9/97.6	31.2	14.4	5.4	0.00	2.09	0.04	0.27	409
	VMAT-SIB	42.8/95.0	29.8	3.4	0.83	0.01	1.11	0.16	0.68	168
4	Helical IMRT-SIB A	43.5/96.6	31.8	10.7	3.4	0.00	6.03	0.05	2.00	480
	Helical IMRT-SIB B	43.4/96.5					0.36	0.06	0.07	
	VMAT-SIB A	43.6/96.8	32.3	16.9	5.6	0.79	8.06	0.13	3.13	195
	VMAT-SIB B	44.9/99.7					1.68	0.12	0.71	
5	Helical IMRT-SIB	44.6/99.1	31.4	4.3	1.55	0.00	1.83	0.02	0.46	485
	VMAT-SIB	44.9/99.8	31.9	3.6	1.34	0.07	3.84	0.06	0.22	237
6	Helical IMRT-SIB	44.2/98.3	31.3	4.0	0.93	0.00	1.69	0.04	0.41	401
	VMAT-SIB	44.9/99.7	31.0	2.6	1.06	0.06	2.08	0.05	0.73	235
7	Helical IMRT-SIB	44.2/98.2	30.5	3.3	1.00	0.00	1.38	0.03	0.34	276
	VMAT-SIB	43.1/95.8	31.3	2.8	0.90	0.00	1.11	0.05	0.00	247
8	Helical IMRT-SIB A	43.8/97.3	31.6	13.6	6.9	0.05	1.77	0.05	0.49	366
	Helical IMRT-SIB B	44.4/98.6					3.57	0.02	0.28	
	VMAT-SIB A	42.3/94.0	29.9	3.5	0.80	0.00	1.11	0.07	0.02	149
	VMAT-SIB B	43.3/96.1					1.58	0.04	0.01	
9	VMAT-SIB	43.3/99.3	30.3	1.5	0.50	0.00	1.19	0.05	0.15	133
	Helical IMRT-SIB	44.7/96.1	30.5	3.0	1.30	0.01	2.45	0.02	0.61	372
10	Helical IMRT-SIB A	44.7/99.3	31.0	8.2	3.6	0.00	2.12	0.02	0.54	384
	Helical IMRT-SIB B	44.5/98.9					1.98	0.03	0.49	
	VMAT-SIB A	40.9/90.9	30.4	2.4	0.45	0.00	0.98	0.12	0.15	267
	VMAT-SIB B	43.3/96.3					1.32	0.05	0.15	
	Helical IMRT-SIB (mean)	44.24	31.15	7.65	3.08	0.02	2.44	0.03	0.52	440.00
	VMAT-SIB (mean)	43.57	30.96	4.32	1.37	0.11	2.15	0.08	0.57	210.10
	p T student	ns	ns	ns	ns	ns	ns	0.000	ns	0.004

Legend: Patients number 4, 8 and 10 had two brain metastases, volume and the dosimetric parameters of each lesion (A and B) are reported in the table

Time of treatment delivery was significantly longer in Helical IMRT plans (either SRS and SIB, Tables 3 and 4). Helical IMRT-SRS fractions lasted 3 times longer than the VMAT SRS ones (1598 s vs 468 s, *p* < 0.001). SIB plans were faster to deliver than the SRS ones but still Helical IMRT lasted twice as long (440 s vs 210 s, *p* = 0.004).

## Discussion

The efficacy and feasibility of a stereotactic boost associated with WBRT has been shown in two randomized clinical trials. In the RTOG 9508 trial [12] 333 patients with 1–3 brain metastases were randomly allocated to either WBRT or SRT-WBRT: WBRT and stereotactic boost treatment improved functional autonomy for all patients and survival for patients with a single metastasis. In the secondary analysis performed after 10 years 252 patients have been re-classified according to the GPA scale and re-analyzed. Survival advantage was found only in patients with high GPA score (3.5-4) regardless of whether they have 1, 2, or 3 brain metastases. A smaller trial [31] showed that the combination of WBRT and radiosurgery for patients with two to four brain metastases significantly improves control of brain disease, without improvement of survival. Logistically, radiosurgery requires separate localization and treatment procedures that add some inconvenience and cost for patients, providers and caregivers. Single fraction treatments also do not permit the exploitation of the potential radiobiologic benefits of re-assortment and re-oxygenation that may occur with a fractionated radiotherapy course. Hall et al. [22] have argued that fractionated stereotactic radiotherapy may be more efficacious in the treatment of neoplastic disease compared to single fraction radiosurgery. Additionally, tumour cell repopulation or sub-lethal damage repair may occur if there is a significant break between the radiosurgery and whole brain radiotherapy sessions. Finally, depending on the radiosurgery system used, treatment of more than 3 metastases may involve prohibitively long treatments, requiring multiple sessions or omission of radiosurgery entirely. The introduction of in-room image guidance systems integrated with radiation treatment machines has led to the introduction of non-invasive stereotactic radiosurgery/radiotherapy techniques. One such unit, Helical IMRT combines intensity modulated fan-beam radiotherapy delivery with megavoltage computed tomography (MVCT) imaging for integrated patient positioning and treatment delivery [32, 33]. From a dosimetric point, the ability to incorporate boost contributions with larger field volumes as part of the treatment planning optimization process provides an advantage of the simultaneous boost strategy over sequential whole brain radiotherapy with radiosurgery boost. This advantage occurs as the radiosurgery boost dose is added to the previously delivered whole brain dose without opportunity for optimization of these two components; the need for two different plans potentially results in unintended increased dose to the brain [34].

In the literature there are numerous studies, in which the use of SIB for the treatment of brain metastases was proposed with different techniques. They are all feasibility studies and, sometimes, dose escalation studies with the aim of reaching doses comparable to those of sequential SRS, according to the linear quadratic cube (LQC) model [20]. The first study was published by Bauman et al. in 2007 [25]: fourteen patients underwent radiotherapy in 10 fraction, 60 Gy on the lesions and 30 Gy on whole brain. They choosed this regimen since the dose of 60 Gy in 10 fractions was equated to 18 Gy in single fraction through the LQC model. The comparison to conventional non-coplanar arc fractionated stereotactic radiotherapy plan demonstrated similar target coverage and improved critical tissue sparing even for a challenging anatomy with multiple lesions in the same plane as the optic apparatus. The Authors concluded that the approach with Helical IMRT has potential advantages such as frameless stereotactic localization through daily megavoltage computed tomography, more efficient use of resources and exploitation of radiobiologic advantages of fractionation. After that other Authors have proposed different fractionation regimes: Weber [34] judged well-tolerated treatments with VMAT in 10 fractions (30 Gy and 40 Gy on whole brain and on metastatic lesions, respectively); Rodrigues [35] confirmed the data of Bauman [25] with 60 Gy in 10 fr on metastases with Helical IMRT, after a dose-escalation study from 35 to 60 Gy in 10 fr; Zhou [36] proposed WBRT in 20 fr with a total dose of 40 Gy and during the last week of treatment a simultaneous boost on brain metastases of 20 Gy in 5 fr with IMRT-IGRT.

The sparing of hippocampal areas, during WBRT with volumetric techniques, had already been studied by Gondi [37] in 2010, with the aim of reducing the impact of WBRT on cognitive functions of long-term survivors patients. Recently the use of the SIB with hippocampal sparing has been the object of a greater interest; the aim is to boost the site of disease without interfering with the neurocognitive functions [38, 39].

In literature dosimetric comparison trials were not published. With the drawbacks of a limited sample size, our work is therefore the first dosimetric comparative study between techniques (Helical IMRT vs VMAT) and boost delivering modality (SRS vs SIB). Although the number of cases of the present series does not allow to draw clinically sound conclusions, the choice of using a panel of descriptors including conformal index, conformal number and homogeneity index has been made to obtain a synthetic evaluation all the dosimetric issues *potentially* influential for the cost/benefit ratio of the treatments delivered with the different techniques.

Only one trial has already made a comparison between the different modality of boost administration. Rodrigues et al. [40] in 2013 published a retrospective review of 500 patients treated with SRS alone or SIB-WBRT: no differences in OS were found but SIB was associated with a reduced intracranial failure rate likely due to the WBRT component of the treatment; however, SRS patients did not have WBRT, at variance with the present series. No dosimetric comparison was performed.

## Conclusions

Although limited and preliminary, this series shows that SRS or SIB BM treatments are feasible with both Helical IMRT and VMAT techniques. A notable dosimetric advantage emerged for Helical IMRT plans, in particular to reduce the normal brain irradiated volume (Brain-PTV) in SRS and other critical organs, such as chiasm and brainstem, in SIB. These clinical findings cannot be generalized due to the exiguous number of cases and heterogeneous locations of lesions. No relevant differences were evident for PTV boost coverage, although VMAT plans are more frequently not optimal, in particular for cases with two BM. Mild discrepancies were presented in terms of dosimetric parameters (slightly in favour of Helical IMRT plans, especially for HI). This assessment may be due to the intrinsic characteristics of Helical IMRT. In this regard is useful to note that the width of MLC leaves is smaller in Helical IMRT machine than in the Linac used (0.6 cm vs 1 cm) and this contributes to increase dose conformation to the target.

A longer treatment delivery time resulted the more important intrinsic limit of Helical IMRT. Too long treatment times should be avoided in order to reduce intrafraction error and patient discomfort.

SIB is a practical way to deliver radiotherapy in BM as an alternative to SRS, since includes WBRT and boost on BM in the same plan. It is therefore characterized by logistic advantages, such as:

- a single simulation, CT-acquisition and elaboration procedure;
- a reduction of waiting times;
- reduced dose delivery time.

All these advantages can be translated in an increased tolerability and treatment compliance for the patient.

In addition, SIB seems to have advantages also from the radiobiological point of view due to the possible reduction of the phenomena of repopulation and redistribution.

In summary, this study suggests that Helical IMRT-SIB is able to deliver slightly better plans when compared to VMAT-SIB, but longer treatment times with Helical IMRT-SRS sometimes prohibitive for patients. In these cases SIB plans should be preferred to improve the global outcome of treatment.

### Ethical approval and consent to participate

Not applicable. Our manuscript does not contain sensitive data from human patients. We performed a retrospective analysis on delivered treatment planning and then we recalculated in silico new treatment planning for each boost delivering and radiotherapy techniques, without affection in real administered treatment. All procedures performed in studies were in accordance with the ethical standards of the institutional and/or national research committee and with the 1964 Helsinki declaration and its later amendments or comparable ethical standards.

### Consent for publication

Not applicable.

### Availability of data and materials

Open access dataset on https://zenodo.org/deposit/92796/.

## Abbreviations

BM: brain metastasis
CI: conformal index
CN: conformal number
Fr: fractions
GPA: graded prognostic analysis
HI: homogeneity index
ICRU: International Commission on Radiation Units & Measurements
KPS: Karnofski performance status
LQ: linear quadratic
LQC: linear quadratic cubic
MLC: multi-leaf collimator
MRI: magnetic resonance
OAR: organ at risk
OS: overall survival
RPA: recursive partitioning analysis
SIB: simultaneous integrated boost
SRS: stereotactic boost
SUR: surgery
TPS: treatment planning system
VMAT: volumetric modulated arc therapy
WBRT: whole brain radiotherapy
